# One-dimensional silver nanostructures on single-wall carbon nanotubes

**DOI:** 10.1186/1556-276X-6-602

**Published:** 2011-11-23

**Authors:** Eunice Mercado, Steven Santiago, Luis Baez, Daniel Rivera, Miguel Gonzalez, Milton E Rivera-Ramos, Madeline Leon, Miguel E Castro

**Affiliations:** 1 Department of Chemistry, Chemical Imaging Center, The University of Puerto Rico at Mayaguez, Mayaguez, 00682, Puerto Rico

## Abstract

We report the synthesis and characterization of one-dimensional silver nanostructures using single-wall carbon nanotubes (SWCNT) as a template material. Transmission electron microscopy and scanning tunneling microscopy are consistent with the formation of a one-dimensional array of silver particles on SWCNT. We observe evidence for the excitation of the longitudinal silver plasmon mode in the optical absorption spectra of Ag-SWCNT dispersions, even in the lowest silver concentrations employed. The results indicate that silver deposits on SWCNT may be candidates for light-to-energy conversion through the coupling of the electric field excited in arrays of plasmonic particles.

## Introduction

There is a worldwide interest in the development of technologies for efficient use and conversion of sunlight into useful energy forms, including heat and electricity. Such technologies promise to result in economic benefits and improvement in the environment. Any rustic and simple energy conversion device must contain a material that absorbs light and converts it into an energy output. Several optical materials may have suitable properties for light absorption and energy conversion, but how to trap and conduct energy over a distance remains a fundamental question.

Electrons and holes have been the choice of charge transport in light-to-energy conversion [1,2]. Electron scattering results in heating devices, but it limits applications that would produce electrical energy. An innovative idea that has emerged in recent years takes advantage of the electric field generated by the excitation of plasmons in nanoparticles. The plasmon frequency corresponds to the energy at which the dielectric constant is zero, and all light is converted into the excitation of a group of electrons and the formation of an electric field. In isolated spherical nanoparticles, only the transverse plasmon mode is excited at the resonance frequency, while the longitudinal mode is readily observed in the optical absorption spectra of nanorods and nanowires [3,4]. Theoretical predictions and recent experimental evidence support the proposal that there is a strong coupling among adjacent nanoparticles that enables the excitation of the longitudinal plasmon mode in particles aligned in one dimension [5,6]. In practice, one-dimensional alignment of nanoparticles is not a simple task and requires a support. In this regard, glass matrices and multiwall carbon nanotubes have been used to study coupling of the nanoparticles and their contribution to the longitudinal mode of the plasmon absorption band [7-11]. We report on the use of single-wall carbon nanotubes (SWCNT) to template one-dimensional silver nanostructures.

**Scheme 1:**
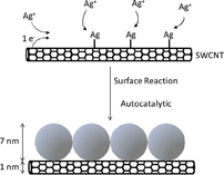
**Deposit of silver on SWCNT**.

Our findings are consistent with the deposition of silver nanoparticles on the SWCNT surface. As illustrated in Scheme 1, the reduction of the silver cations present in solution by the electron rich SWCNT results in the deposition of silver on the SWCNT surface. Further absorption of silver cations from the solution results in the formation of nanoparticles in close proximity to each other. Transmission electron microscopy (TEM) and scanning tunneling microscopy (STM) measurements of SWCNT with the lowest silver loads are consistent with the formation of discrete silver-rich regions on the nanotubes.

We observe evidence for the optical excitation of the longitudinal silver plasmon mode, even with the lowest silver concentrations employed, a result consistent with simulations of light absorption by continuous one-dimensional nanostructures. The results encourage further research on the use of SWCNT as templates for the development of nanostructured plasmonic devices for the light to electrical energy conversion.

## Experimental section

### Materials

The single-wall carbon nanotubes employed for the work described here were purchased from Cheap Tubes Inc (Brattleboro, VT, USA). The silver nitrate (AgNO_3_) used in the silver nanoparticle synthesis and the ethylene glycol used as a solvent in our experiments were obtained from Sigma-Aldrich and used without further purification.

### Equipment

UV-visible absorption measurements were conducted using an Agilent Spectrophotometer model 8453 (Biodirect, Inc., Taunton, MA, USA). A quartz cuvette with an optical path of 0.25 cm was used for the optical absorption measurements. Scanning tunneling microscopy (STM) measurements were performed in a NanoSurf Easy Scan E-STM (Nanosurf Inc., Boston, MA, USA), version 2.1, using a Pt/Ir tip. The STM was calibrated with measurements performed on a commercial gold ruler. Measurements performed on longitudinal features of dry deposits of submonolayer quantities of C_12_-SH and C_10_-SH alkyl thiols coincided with the expected molecular lengths of these molecules. A drop of the silver/SWCNT dispersion was deposited on a highly oriented graphite attached to a magnetic holder and allowed to dry prior to the measurements. TEM measurements were performed with a JEOL 2010 electron microscope (JEOL USA, Inc., Peabody, MA, USA). The samples were outgassed at 10^-3 ^Torr for several days prior to placement in the TEM sample compartment. TEM measurements were performed with an acceleration voltage of 120 kV. Negatives of the micrographs were processed using standard techniques and scanned with an EPSON Perfection V750 PRO scanner (Epson, Long Beach, CA, USA) and stored in a PC computer for further analysis. Scanning electron microscopy measurements were performed with a JEOL 6460 LV SEM instrument (JEOL USA, Inc., Peabody, MA, USA) equipped with an X-ray detector for energy dispersive X-ray spectroscopy (EDAX) measurements.

### Silver nanoparticles synthesis

A 1 × 10^-2 ^M AgNO_3 _solution was prepared using ethylene glycol as solvent. Two subsequent aliquots were used to prepare 5 ml of 1 × 10^-3 ^and 1 × 10^-5 ^M silver solutions. A quantity of 0.0023 g of SWCNT was added to each solution which was then warmed to 470 K. The solutions were used to obtain the UV-visible measurements 24 h later. SEM and EDAX measurements were obtained from the solution with the highest silver concentration, 1 × 10^-2 ^M. The formation of silver nanoparticles templated on SWCNT resulted from the solution with the lowest silver concentration, 1 × 10^-5 ^M. A dry deposit of the solution was analyzed by TEM and STM techniques.

### Computer simulations

Simulations of the optical absorption spectra of silver spheres are based on Mie theory. The wavelength-dependent absorbance (*A*) of light by a substance is given by:

(1) $$A=n\gamma I_{\text{o}}∕\ln10$$

where *n *represents the number of absorbers, *γ *is the absorption cross section, and *I*_o _is the incident light intensity. For spheres smaller than the wavelength of the incident light, the absorption cross section may be estimated by calculating the dipole contribution to the absorption spectra as:

(2) $$\gamma \;=\;9{\varepsilon_{\alpha }}^{3∕2}V\;\left(\omega ∕c\right)\varepsilon_{2}∕\left\{{\left[\varepsilon_{1}\;+2\;\varepsilon_{2}\right]}^{2}+\;{\varepsilon_{2}}^{2}\right\}$$

where *ε*_*α *_is the dielectric constant of the medium, *ω *is the frequency of the incoming radiation, *c *is the speed of light, and *ε*_1 _and *ε*_2 _represent the real and imaginary parts of the particle's dielectric constant (*ε*). In the case of silver, the real and imaginary parts of the dielectric constants have contributions from interband transitions (IB) and the excitation of the plasmon (P):

(3) $$\begin{array}{cc} \varepsilon_{\text{1}}=\varepsilon_{\text{1IB}}+\varepsilon_{\text{1},\text{P}} & \varepsilon_{\text{2}}=\varepsilon_{\text{2IB}}+\;\varepsilon_{2\text{P}} \end{array}$$

The plasmon contributions to the components of the dielectric constant are calculated as:

(4) $$\begin{array}{cc} \varepsilon_{1}=1-{\omega_{\text{P}}}^{2}∕\left(\omega^{2}+{\omega_{\text{o}}}^{2}\right) & \varepsilon_{2}={\omega_{\text{p}}}^{2}\omega_{\text{o}}∕\left[\omega \left(\omega^{2}+{\omega_{\text{o}}}^{2}\right)\right] \end{array}$$

where *ω*_P _and *ω *represent the frequencies corresponding to the bulk plasmon and incident light, and *ω*_o _is the size-dependent surface scattering rate estimated as:

(5) $$\omega_{\text{o}}=Av_{\text{F}}∕r$$

where *A *is proportionality factor, *v*_F _is the Fermi velocity, and *r *is the particle radii.

The simulations of the absorption spectra of the one-dimensional structures are based on the Gans treatment of Mie theory. The absorption cross section within the dipole approximation is calculated as:

(6) $$\frac{\gamma }{N_{P}V}=\frac{2\Pi \in_{\alpha }^{1/2}}{3\lambda }\underset{j}{\sum }\frac{\left(\frac{1}{P_{j}^{2}}\right)\in_{2}}{{\left[\in_{1}+\left(\frac{1-P_{j}}{P_{j}}\right)\in_{\alpha }\right]}^{2}+\in_{2}^{2}}$$

where *N*_P _and *V *represent particle concentration and volume, respectively, and *λ *is the incident light wavelength. The contributions of the real (*ε*_1_) and imaginary (*ε*_2_) components of the refractive index are obtained from Harris et al. [10]. In the equation, *P_j _*represents a geometric factor related to the coordinates of an elliptical particle [12]. The letters used in the *P_j _*represent the longitudinal axis "A" and transverse axes "B" and "C." In elongated ellipsoids, B and C are equal and represent the diameter (*d*) of the ellipsoid.

## Results and discussion

### UV-visible absorption measurements

**Figure 1:**
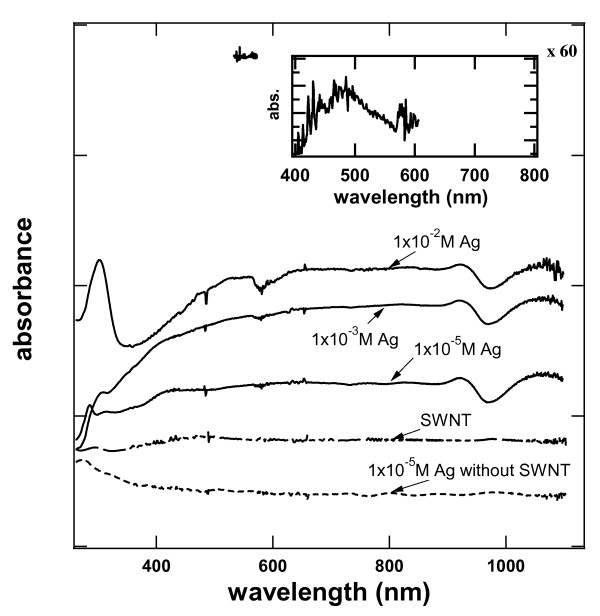
**The UV-visible spectra of Ag-SWCNT dispersions**. As a function of [AgNO_3_] between 250 and 850 nm. Representative spectra of the SWCNT and [AgNO_3_] solutions employed in the work are indicated in the figure.

Figure 1 summarizes the absorption spectra of the SWCNT dispersions warmed in the presence of different AgNO_3 _concentrations. For reference, the spectrum of a AgNO_3 _solution in the absence of the SWCNT is also indicated. The optical absorption spectrum of the AgNO_3 _solution does not exhibit any significant absorption features above 400 nm. The absorption spectrum of the SWCNT dispersions employed for the experiments reported here are also indicated in the same figure.

The optical absorption spectrum of the SWCNT dispersion does not exhibit significant absorption above 400 nm, although considerable fine structure can be observed within the noise level of the measurement. The insert in Figure 1 shows the optical absorption spectra for of the SWCNT dispersion between 400 and 800 nm multiplied by a factor of 60 to adjust it to the scale displayed with the other data. This fine structure is not noise as it is not observed in measurements of the solvent, cell, or air performed in the same instrument under otherwise identical experimental conditions. The absorption and emission spectra of carbon nanotubes have been the subject of various studies [13,14]. Light absorption is a response of the electronic properties and structure of SWCNT corresponding to metallic, semi-metallic, and semiconducting structures. Fine structure has been documented in isolated carbon nanotubes or dispersions of SWCNT [15,16]. When the carbon nanotubes are not dispersed, electronic coupling mixes energy states among different SWCNT in a bundle, limiting the observation of fine structure. The SWCNT used in this experiment consist of 60% semi-metallic and 40% metallic structures. While we are not able to spot bands characteristic of individual SWCNT, the fine structure observed is consistent with the formation of SWCNT dispersion in ethylene glycol.

The spectrum of the SWCNT dispersion is significantly affected by the presence of the AgNO_3 _in solution. The spectra of different Ag-SWCNT dispersions for three different AgNO_3 _concentrations are indicated in the same figure. Ag-SWCNT dispersion spectra are characterized by well-defined absorption features around 300 nm and a broad absorption band that starts around 400 nm and extends well above 800 nm. The absorption of the Ag-SWCNT dispersion increases with the AgNO_3 _load. Optical absorption measurements on AgNO_3 _solutions at room temperature or warmed to 470 K without the SWCNT did not exhibit significant absorption in visible wavelengths. Thus, the observed optical absorption spectrum is attributed to the deposition of silver on the SWCNT surface.

### Simulations of absorption spectra of spheres and elongate structures

**Figure 2:**
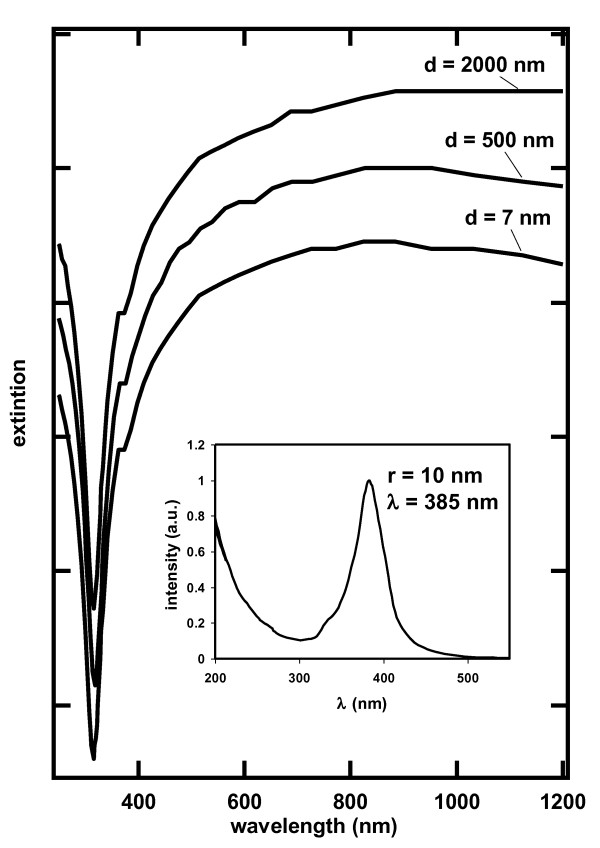
**Dependence of the absorption cross section**. As a function of wavelength for one-dimensional silver structures with the indicated diameters (*d*) and a length of 2,500 nm. The insert illustrates the absorption cross section of 10-nm silver spheres.

Figure 2 illustrates simulations of the dependence of *γ *as a function of wavelength for elongated one-dimensional silver structures. For reference, the result of a simulation on a 10-nm silver sphere is illustrated on the figure. The spectrum is characterized by a band around 385 nm resulting from the excitation of the transverse plasmon mode in the spheres and a short wavelength tail that results from interband transitions.

The contribution of the longitudinal plasmon mode to the optical absorption spectrum is readily observed in the simulations corresponding to elongated silver nanostructures. The structures considered for the simulation have a length of 2,500 nm and diameters of 7, 500, and 2,000 nm. The structure of the absorption spectra is nearly insensitive to the diameter of the elongated nanostructure although the amount of light absorbed increases with the diameter of the structure at all wavelengths. The amount of light absorbed increases with wavelengths above 300 nm and extends to the near infrared in the spectra of the three elongated structures considered. The trend in light absorption toward long optical frequencies in elongated nanostructures is in marked contrast with those observed in spherical particles, a difference that results largely from the excitation of the longitudinal plasmon mode in the former nanostructures [12]. The extraordinary resemblance of the spectra discussed above with those predicted by the simulation displayed in Figure 2 lead us to conclude that the optical absorption spectra of the Ag-SWCNT dispersion results from the formation of one-dimensional silver structures on the SWCNT.

### Characterization of Ag-SWCNT dispersions

**Figure 3:**
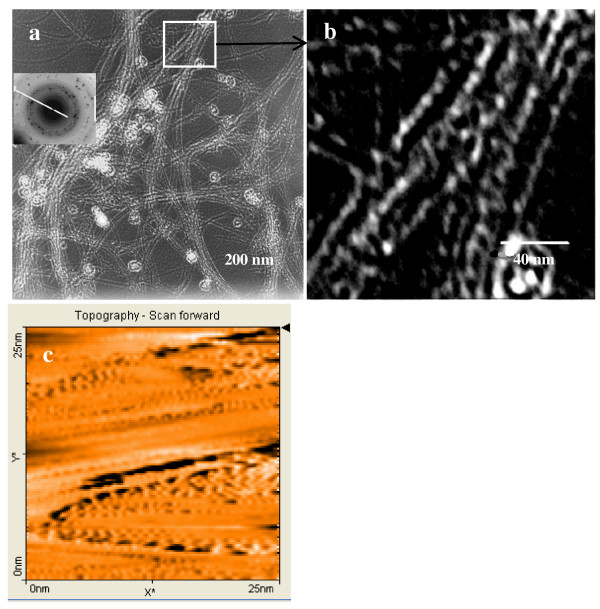
**TEM images and STM image of Ag-SWCNT assemblies**. The AgNO_3 _concentration used for the preparation of the dispersion is 1 × 10^-5 ^M.

Representative TEM and STM images of a dry deposit of the 1 × 10^-5 ^M Ag-SWCNT dispersion are displayed in Figure 3. Well-dispersed SWCNT are readily identified in Figure 3a, consistent with the fine structure discussed in the context of the UV-visible absorption spectrum of the silver-SWCNT dispersion. Silver particles, about 30 nm in diameter, are formed while focusing the electron beam on the carbon grid used to support the sample. The diffraction pattern displayed on the inset of Figure 3a is consistent with an arrangement of polycrystalline silver atoms in the particle [17]. Figure 3b corresponds to the region in Figure 3a enclosed with a square. Particles that are about 7 nm in diameter, about three times the diameter of the 1.9-nm SWCNT, are readily observed. STM measurements of deposits prepared from the same 1 × 10^-5 ^M Ag-SWCNT dispersion are displayed on Figure 3c. The STM images are consistent with the formation of one-dimensional silver nanostructures from the alignment of particle-like structures.

**Figure 4:**
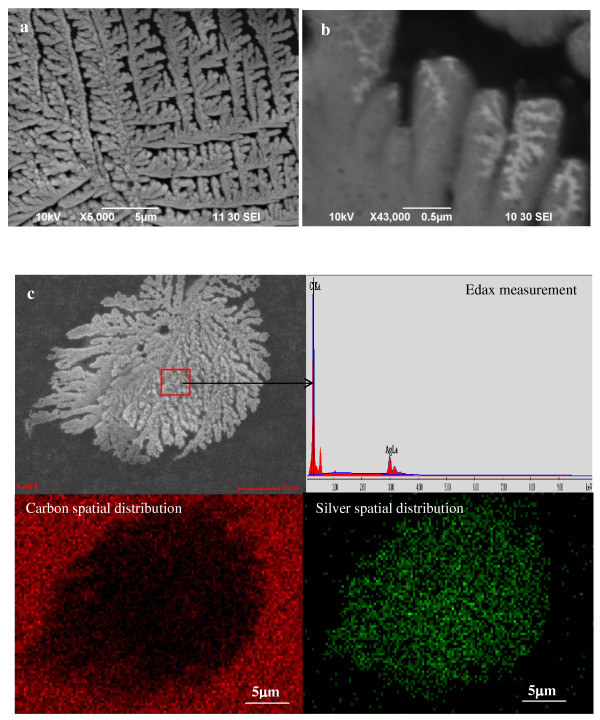
**SEM and EDAX mapping measurement images of a Ag-SWCNT dispersion**. With a AgNO_3 _concentration of 1 × 10^-2 ^M.

Dry deposits from samples with a larger silver content resulted in the formation of structures that required the use of the SEM for appropriate imaging. Figure 4 illustrates representative images of measurements performed on dry deposits of the Ag-SWCNT dispersions with an initial silver concentration of 1 × 10^-2 ^M. The formation of dendrite-like structures shown in Figure 4a was common in the deposit. The smallest roughness features that we can spot in the image are shown in Figure 4b and have dimensions in the order of about 20 nm. Figure 4c shows EDAX mapping measurements of the same sample. It reveals well-defined regions containing silver, consistent with the deposition of silver on the SWCNT.

## Discussion

The imaging measurements performed on the SWCNT dispersions are consistent with the formation of one-dimensional silver nanostructures. The absorption spectra of all the Ag-SWCNT dispersions reported here have a similar structure, characterized by a significant increase in light absorption as the wavelength increases from UV to visible due to the excitation of the longitudinal plasmon mode. The simulations summarized in Figure 2, consistent with the experimental UV-visible absorption measurements, are consistent with the excitation of the longitudinal mode of silver nanostructures. Small differences between the simulated and experimental spectra rise likely rise from difference in the details of the morphology of the nanostructure: these differences are more notably between 300 and 400 nm, probably reflecting a small contribution arising from the transverse plasmon mode in silver. The simulations of the UV-visible absorption were performed on a silver film that is continuous in one dimension. The experimental evidence, particularly the TEM and STM images displayed on Figure 3, are consistent with the formation of discrete silver regions - about 7 nm wide - on the SWCNT surfaces. Electromagnetic coupling among these silver regions formed on the SWCNT surfaces could explain the observed light absorption spectra reported here. Sweatlock and coworkers performed theoretical calculations with the objective of establishing the contribution of the longitudinal plasmon mode to the absorption spectrum of one-dimensional arrays of 4, 8, and 12 spherical silver nanoparticles [18]. They reported that the longitudinal plasmon band shifted toward longer wavelengths with increasing the number of particles in a one-dimensional arrangement. Next neighbor distance was found to play an important role in the predicted longitudinal plasmon absorption band, which was found to be inversely related to the particle-to-particle distance. Pinchuk and Schatz performed calculations on one-dimensional arrays of silver nanoparticles [19]. They found that the coupling of the electromagnetic field among silver nanoparticles arranged in one-dimensional arrays is sensitive to the particle-to-particle distance resulting in a broadening of the absorption band. Enoch et al. found that a small change in interparticle distance is enough to make a significant change in the absorption spectra: changes in interparticle distance smaller than 4 nm result in a red shift of the plasmon absorption band and a broadening of the absorption spectrum [20]. Near-field coupling of the electromagnetic field has also been reported by Maier [21], who found the dipole model adequate for electromagnetic energy transfer below the diffraction limit in chains of closely spaced metal nanoparticles. The spatial distribution of nanoparticles was found to play a role in electromagnetic coupling and the plasmon resonance band [22] Unfortunately, we cannot establish a separation among these silver regions in a given nanotube from our measurements: in fact, the silver regions appear to be in contact in the STM image displayed on Figure 3c.

It could be argued that plasmonic nanoparticles also affect the optical properties of the carbon nanotubes. Indeed, Hanson has predicted that the presence of a plasmonic nanoparticle on a carbon nanotube wall affects the electric field and current on the carbon nanotube, and can be used to induce relatively large currents on the tube in the neighborhood of the sphere [23]. This view is consistent with recent experimental work. Sakashita reported the enhancement of photoluminescence intensity of single carbon nanotubes coupled to a rough gold surface. It was attributed to local field enhancement of the incident light induced by localized surface plasmons [24]. However, the effect of plasmonic nanoparticles on the optical properties on SWCNT results in localized absorption in the neighborhood of the nanoparticle absorption plasmon wavelength, as opposed to the rather broad absorption spectra resulting from the excitation of the longitudinal plasmon mode observed here. In the case of silver nanospheres, the transverse mode is located between 300 and 400 nm. The significant structure found in the absorption spectra around 300 nm may result from the coupling predicted by Hansen, but further experimental work is necessary to establish the effect of plasmonic nanoparticles on the optical absorption spectrum of the SWCNT.

## Summary

In summary, we have used single-wall carbon nanotubes (SWCNT) to template one-dimensional silver nanostructures. We observed evidence of the excitation of the longitudinal silver plasmon mode in the optical absorption spectra of Ag-SWCNT dispersions, even at the lowest silver concentrations employed. Tunneling and electron microscopy measurements are also consistent with the formation of one-dimensional silver nanostructures. The results indicate that silver deposits on SWCNT may be suitable candidates for light-to-energy conversion through coupling of the electric field excited in plasmonic particles.

## Competing interests

The authors declare that they have no competing interests.

## Authors' contributions

EM and MEC made the analysis and interpretation of the data and draft the manuscript. SS and LB helped with the literature review and along with DR carried out the STM measurements. MER participated in the acquisition of the data for the SEM experiments and, along with ML, revealed the negatives of the micrographs of the TEM experiments. ML and MG helped with the TEM measurements and data interpretation. MC helped with the data interpretation and the preparation of the manuscript.
